# TOPK modulates tumour-specific radiosensitivity and correlates with recurrence after prostate radiotherapy

**DOI:** 10.1038/bjc.2017.197

**Published:** 2017-07-04

**Authors:** Giacomo Pirovano, Thomas M Ashton, Katharine J Herbert, Richard J Bryant, Clare L Verrill, Lucia Cerundolo, Francesca M Buffa, Remko Prevo, Iona Harrap, Anderson J Ryan, Valentine Macaulay, William G McKenna, Geoff S Higgins

**Affiliations:** 1 CRUK/MRC Oxford Institute for Radiation Oncology, University of Oxford, Old Road Campus Research Building, Roosevelt Drive, Oxford OX3 7DQ, UK; 2 Nuffield Department of Surgical Sciences, Oxford Cancer Research Centre, University of Oxford, Old Road Campus Research Building, Roosevelt Drive, Oxford OX3 7DQ, UK

**Keywords:** PBK, TOPK, radiosensitivity, cancer

## Abstract

**Background:**

Tumour-specific radiosensitising treatments may enhance the efficacy of radiotherapy without exacerbating side effects. In this study we determined the radiation response following depletion or inhibition of TOPK, a mitogen-activated protein kinase kinase family Ser/Thr protein kinase that is upregulated in many cancers.

**Methods:**

Radiation response was studied in a wide range of cancer cell lines and normal cells using colony formation assays. The effect on cell cycle progression was assessed and the relationship between TOPK expression and therapeutic efficacy was studied in a cohort of 128 prostate cancer patients treated with radical radiotherapy.

**Results:**

*TOPK* knockdown did not alter radiation response in normal tissues, but significantly enhanced radiosensitivity in cancer cells. This result was recapitulated in TOPK knockout cells and with the TOPK inhibitor, OTS964. TOPK depletion altered the G_1_/S transition and G_2_/M arrest in response to radiation. Furthermore, TOPK depletion increased chromosomal aberrations, multinucleation and apoptotic cell death after irradiation. These results suggest a possible role for TOPK in the radiation-induced DNA damage checkpoints. These findings have clinical relevance, as elevated TOPK protein expression was associated with poorer clinical outcomes in prostate cancer patients treated with radical radiotherapy.

**Conclusions:**

This study demonstrates that TOPK disruption may cause tumour-specific radiosensitisation in multiple different tumour types.

Radiotherapy has a major role in cancer treatment. Increasing ionising radiation (IR) dose may improve the probability of local tumour control but increases the risk of damage to the surrounding healthy tissue. The ‘therapeutic window’ is the range of radiation doses that can be delivered to patients with limited side effects.

Intrinsic radiosensitivity gives rise to differences in susceptibility to ionising radiation (IR) treatment and impacts on clinical endpoints, including survival, local control and disease-free survival (Hanahan and Weinberg, 2011). Our laboratory developed a high-throughput colony formation assay (CFA) and screened an siRNA library of 709 protein kinases (Tiwana *et al*, 2015), which identified T-LAK cell-originated protein kinase, *TOPK* (also known as PDZ-binding kinase) as a highly ranked radiosensitising gene. We chose to further investigate TOPK as a potential target to widen the therapeutic window due to its differential expression between cancer and normal tissues.

TOPK is a Ser/Thr protein kinase of 322 amino acids first identified from a lymphokine-activated killer T-cell subtraction cDNA fragment library (Abe *et al*, 2000) and as a physical interactor of the hDlg tumour suppressor protein (Gaudet *et al*, 2000). TOPK is a member of the mitogen-activated protein kinase kinase family involved in growth, migration and mitotic progression (Abe *et al*, 2000; Gaudet *et al*, 2000; Matsumoto *et al*, 2004; Shih *et al*, 2012; Shinde *et al*, 2013; Park *et al*, 2006). TOPK activity is regulated by CDK1/cyclin B-mediated phosphorylation at its Thr9 residue, which promotes mitotic entry at the G_2_/M checkpoint (Abe *et al*, 2000; Gaudet *et al*, 2000; Matsumoto *et al*, 2004). TOPK inhibits PTEN, leading to AKT/PI3K activation (Shih *et al*, 2012; Shinde *et al*, 2013). During mitosis, TOPK phosphorylates histone H3 (Ser10) during the transition from prophase to metaphase, (Park *et al*, 2006) specifically targets C2H2 zinc finger proteins to promote chromatin dissociation (Rizkallah *et al*, 2015) and complexes with cyclin B/CDK1 to phosphorylate PRC1 and promote chromosomal separation and cytokinesis (Abe *et al*, 2007). TOPK depletion results in cytokinetic failure and cell cycle arrest, (Abe *et al*, 2007; Park *et al*, 2010), which leads to multinucleation in HeLa cells (Matsumoto *et al*, 2004). TOPK directly inhibits the tumour-suppressor p53 with subsequent downregulation of p21 expression and impaired regulatory control over cellular growth (Zykova *et al*, 2010).

TOPK is involved in the cellular response to doxorubicin (Nandi *et al*, 2007; Zykova *et al*, 2010), arsenite (As^3+^) (Zykova *et al*, 2006) and ultraviolet radiation (Ayllon and O'Connor, 2007), but has not been studied in the context of IR. Overexpression of TOPK in HT1080 cells following doxorubicin treatment suppresses G_2_/M checkpoint control, leading to premature mitotic entry and polyploidy (Nandi *et al*, 2007). Furthermore, TOPK co-localises with γ-H2AX foci after arsenite treatment and inhibits apoptosis in RPMI-7951 cells (Zykova *et al*, 2006), and TOPK depletion reduces the number of γ-H2AX foci in MCF-7 cells following UV-induced DNA damage (Ayllon and O'Connor, 2007).

*TOPK* expression is upregulated in most cancers (Rhodes *et al*, 2007); however, expression is restricted in normal tissues to a few organs such as testis and placenta (Abe *et al*, 2000; Gaudet *et al*, 2000; Matsumoto *et al*, 2004; Fujibuchi *et al*, 2005). High *TOPK* expression has been associated with adverse effects on local tumour control in non-small-cell lung cancer and with mutant p53 expression levels (Lei *et al*, 2013; Shih *et al*, 2012; Lei *et al*, 2015). Similarly, high *TOPK* expression correlates with worse overall survival and recurrence-free survival in breast, gastric and colorectal cancer (O'Leary *et al*, 2013; Xiao *et al*, 2015; Ohashi *et al*, 2017). In colorectal cancer, increased levels of TOPK are correlated with high levels of interleukin-8 (Xiao *et al*, 2015) and with oncogenic *KRAS* and *BRAF* mutations (Zlobec *et al*, 2010). In prostate cancer, high *TOPK* expression correlates with increased invasiveness and cancer stage (Sun *et al*, 2015). Conversely, low *TOPK* expression is predictive of poor prognosis in cholangiocarcinoma patients (He *et al*, 2010).

In the present study we report a novel role for TOPK as a tumour-specific modulator of radiosensitivity. TOPK has the potential to be exploited as a clinical radiosensitiser due to its differential expression between cancer and normal tissues. TOPK depletion impairs G_2_/M checkpoint activation and increases chromosomal aberrations, apoptosis and multinucleation. Elevated TOPK protein expression is associated with poorer clinical outcomes in a cohort of prostate cancer patients treated with radical radiotherapy.

## Materials and methods

### Cell culture

HCT116, HeLa, MRC5, HFL-1, DU145, PC3, H1299 and T24 cell lines were purchased from American Type Culture Collection (ATCC, Manassas, VA, USA). SQ2OB cells were provided by Dr Ralph Weichselbaum (University of Chicago, Chicago, IL, USA). T24 cells were maintained in RPMI-1640, MRC5 cells in MEM and HFL-1 cells in DMEM/F-12 Ham’s. The HAP1 cell lines were purchased from Horizon Discovery (Cambridge, UK) and maintained in IMDM medium. HAP1 is a near-haploid human cell line derived from chronic myelogenous leukaemia cell line. HAP1 cells are adherent with fibroblast-like morphology. *TOPK* knockout was achieved via Crispr/Cas9 editing to contain a 4 bp deletion in the coding exon of *TOPK*. HUVEC and HMEC_1 cells were maintained in EBM-Plus (Lonza, Walkersville, MD, USA) medium. All other cells were maintained in DMEM. All cells were maintained with 10% FBS and all medium was purchased from Sigma (St Louis, MI, USA). All cell lines were authenticated by LGC standards (ATCC) by short tandem repeat profiling and tested for mycoplasma using MycoAlert (Lonza).

### siRNA transfection

Reverse-transfection protocol was used (final concentration 20 nM) using Ambion Silencer Select siRNA (LifeTechnologies) INTERFERin-HTS (Polyplus) transfection reagent as previously described (Tiwana *et al*, 2015). si*TOPK*: 5′-GACUAAUGGAUGAAGCUAAtt-3′ si*TOPK*_2: 5′-CCCUGAGGCUUGUUACAUUtt-3′ si*TOPK*_3: 5′-GCACUAAUGAAGACCCUAAtt-3′.

### Cell synchronisation

Cell synchronisation in G_1_/S phase was achieved using 2 mM thymidine block for 18 h, followed by 9 h release in fresh medium and then 14 h further incubation with 2 mM thymidine. Cells were released in fresh medium after two washes in PBS.

### Immunoblotting

Protein lysates were prepared using RIPA lysis buffer (Thermo Scientific, Rockford, IL, USA) with protease inhibitors (Roche, Mannenheim, Germany) and phosphatase inhibitors (Sigma). Protein concentration was determined using the BCA assay (Thermo Scientific). Bound antibodies were detected by developing film from nitrocellulose membranes exposed to chemiluminescence reagent (Immobilon Western Chemiluminescent Substrate, EMD Millipore, Merck KGaA, Darmstadt, Germany).

### Antibodies

The following antibodies were used: anti-TOPK for immunoblotting (Sigma SAB5300406 clone 2C8, 1 : 1000); anti-TOPK for immunohistochemistry (Cell Signaling Technology, Danvers, MA, USA 4942, 1 : 100); anti-TOPK (phospho T9) antibody (Abcam, Cambridge Science Park, Cambridge, UK ab184953, 1 : 1000); anti-cyclin E1 (Cell Signaling Technology 4129, 1 : 1000); anti-cyclin B1 (Cell Signaling Technology 4138, 1 : 1000); anti-phospho-CDK1 (Tyr15) (Cell Signaling Technology 4539, 1 : 1000); anti-CDK1 (Cell Signaling Technology 9116, 1 : 1000); anti-phospho histone H3 (Ser10) (Cell Signaling Technology 3377, 1 : 1000); anti-histone H3 (Cell Signaling Technology 9715, 1 : 1000); anti-Akt (Cell Signaling Technology 9272S, 1 : 1000); anti-phospho-Akt (Cell Signaling Technology 4060S, 1 : 1000); anti-Vinculin (Abcam ab18058, 1:40 000); and anti-γ-H2AX Ser139 (Upstate/Millipore, 05-636, Temecula, CA, USA).

### Colony formation and viability assays

Inhibitor-treated and siRNA-transfected CFAs were performed and analysed as previously described (Tiwana *et al*, 2015). To determine the effect of OTS964 on cell viability, resazurin was added to the cells for 3 h after 72 h drug treatment. Fluorescence was measured using a POLARstar OMEGA plate reader (BMG Labtech, Ortenberg, Germany). IC_50_ was calculated from non-linear curve fitting of Log[Inhibitor] vs Response using variable slope (four parameters; GraphPad Prism Software v 7.0c, San Diego, CA, USA).

### Flow cytometry

Bromodeoxyuridine (BrdU) (20 μM; Sigma) was added to adherent cells 30 min before fixing. Cells were fixed in ice-cold 70% ethanol, incubated at room temperature with 2 M HCl containing 0.1 mg ml^−1^ pepsin for 20 min and resuspended in 100 μl of 2% FBS/1 × PBS with 1 μl mouse anti-BrdU monoclonal antibody (1 : 100, BD Bioscience, San Jose, CA, USA) for 90 min. Samples were resuspended in 100 μl 2% FBS/1 × PBS with 0.5 μl Goat anti-Mouse AF488 antibody (Life Technologies, Eugene, OR, USA) for 60 min in the dark. Samples were washed in PBS and resuspended in 0.5 ml PBS containing 50 μg ml^−1^ propidium iodide (PI). Samples were analysed using FACSort cytometer (Becton Dickson, San Jose, CA, USA).

### Microscopy

For live cell analysis, cells were stably transfected with the pH2B_mCherry_IRES_puro2 plasmid (Steigemann *et al*, 2009) (from Daniel Gerlich, Addgene plasmid 21045). Phase-contrast and fluorescence images were taken every 30 min for 48 h, with cells maintained at 5% CO_2_, 37 °C. For fixed cell analysis, cells were plated onto glass coverslips, fixed with 70% ice-cold methanol for 10 min and then incubated with PBS containing 1 μg ml^−1^ DAPI and 1 μg ml^−1^ FITC. Coverslips were then washed and mounted on a microscope slide using antifade medium Vectashield (Burlingame, CA, USA). Samples were analysed with a confocal microscope (Zeiss710, Zeiss, Oberkochen, Germany) and cell morphology was scored manually using ImageJ open source software. Mitotic chromosomes were captured by incubating the cells in colcemid (30 ng ml^−1^) for 1 h and metaphase nuclei prepared by incubating samples in hypotonic solution (75 mM KCl) for 30 min before fixation in Carnoy solution (3 : 1 methanol:glacial acetic acid). Chromosome suspensions were dropped onto slides and mounted using Vectashield with DAPI. Samples were imaged with × 100 oil-immersion lens using a Nikon 90i confocal microscope (Nikon, Minato, Tokyo, Japan).

### Patient samples

Prostate cancer samples and associated anonymous follow up data (ORB ethics 09/H0606/5+5) included 128 patients who received radical radiotherapy with curative intent. All patients that participated in the study and whose samples were used for analysis provided informed signed consent. Prostate biopsies were collected at the time of diagnosis. External beam radiotherapy was 3D conformal CT planned. A 55 Gy dose was typically delivered to the planned target volume in 20 fractions over a 4-week period with neoadjuvant and concurrent androgen deprivation therapy. Assuming an α/β ratio for prostate cancer of 1.8 Gy (Dearnaley *et al*, 2016), this dose/fractionation schedule is equivalent to 65.9 Gy in 2 Gy fractions. All patients were treated at the Oxford Cancer Centre and human tissue was used with National Research Ethics approval (study 07/H0606/120).

### Immunohistochemistry

Sections were de-paraffinised in histoclear and rehydrated through graded ethanol to water. Endogenous peroxidase activity was inactivated using 3% H_2_O_2_ in methanol. Sections were blocked with 5% normal goat serum and incubated with primary antibody at 4 °C overnight. A biotinylated secondary antibody was added for 45 min followed by an avidin/biotin-based peroxidase solution and incubated with 3,3′-diaminobenzidine solution before counterstaining with haematoxylin. All sections were dehydrated using increasing percentages of ethanol followed by histoclear and mounted in omnimount medium.

### Scoring and statistical analysis

Patient samples were assigned a blind intensity score ranging from 0 (no expression) to 3 (maximal expression) multiplied by the percentage of stained cells (total TOPK expression score, 0–600). For univariate analysis, data were initially binarised as described in the text, whereas significance was assessed using the log-rank test. Cox survival analysis was performed. Analyses were done using SPSS statistic 22 software (UNICOM Global, Mission Hills, CA, USA). All statistical tests were performed as two-tailed tests and differences were considered significant at a *P*-value of less than 0.05. All assay data are representative of three independent experiments and are presented as mean ± s.d. from triplicate wells, unless otherwise stated (*P<0.05, **P<0.01 and ***P<0.001). Survival curves were fitted using nonlinear regression and were analysed by factorial two-way ANOVA, with interaction term significance of *P*<0.05.

## Results

### TOPK is a tumour-specific novel modulator of radiosensitivity

**Figure 1:**
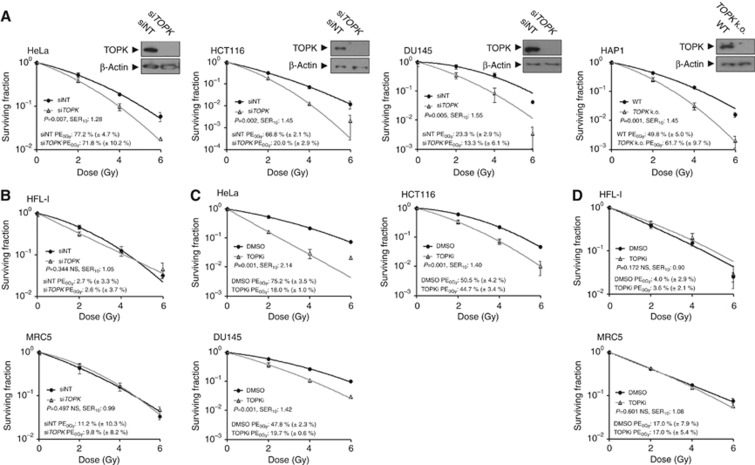
***TOPK* is a tumour-specific modulator of radiosensitivity.**(**A**) *TOPK* was transiently knocked down with siRNA (si*TOPK*) and post-irradiation clonogenic survival was assessed in cancer-derived cells. Transfection with siNT was used as a control and knockdown efficiency was confirmed by immunoblotting (insets). (**B**) No significant TOPK-dependent radiosensitisation was detected in HFL-1 and MRC5 cells. (**C**) TOPK activity was inhibited by 4 h pretreatment with 70–200 nM OTS964 (70 nM for HCT116, DU145, 100 nM for HeLa, 200 nM for HFL-1, MRC5). (**D**) No significant TOPK-dependent radiosensitisation was detected in HFL-1 and MRC5 cells treated with OTS964. All data are representative of three independent experiments and are presented as mean ± s.d. from triplicate wells. Survival curves were fitted using non-linear regression. Results were analysed by factorial two-way ANOVA, with interaction term significance of *P*<0.05. PE=plating efficiency; SER_10_=survival enhancement ratio at a surviving fraction of 0.10.

The impact of *TOPK* knockdown on radiosensitivity was investigated in a panel of cancer cell lines using a CFA. Transient knockdown of *TOPK* was achieved by siRNA transfection at a final concentration of 20 nM (si*TOPK*), with non-targeting siRNA (siNT) used as a control. Radiosensitisation was observed in all siRNA treated cancer cell lines, with the survival enhancement ratio at a SER_10_ of 1.28 (*P*=0.007) in HeLa cells (cervical), 1.45 (*P*=0.002) in HCT116 cells (colorectal), 1.55 (*P*=0.005) in DU145 cells (prostate) and 1.45 (*P*<0.001) in HAP1 *TOPK* CRISPR/Cas9 knockout cells (Figure 1A). Four other cancer cell lines showed similar enhancement ratios: 1.37 (*P*=0.006) in T24 cells (bladder), 1.28 (*P*=0.003) in H1299 cells (lung), 1.13 (*P*<0.001) in SQ20B cells (head and neck) and 1.35 (*P*=0.004) in PC3 cells (prostate; Supplementary Figure 1A).

The Oncomine 3.0 database revealed that *TOPK* expression is predominantly limited to cancer tissues, with many studies showing high *TOPK* expression in tumour types including the brain, breast and lung (Supplementary Figure 1B; Rhodes *et al*, 2007). This is in accordance with the undetectable *TOPK* expression in MRC5 and HFL-1 normal lung fibroblasts (Supplementary Figure 1C). Correspondingly, no significant radiosensitisation was observed in HFL-1 or MRC5 cells, suggesting that the observed radiosensitising effect is tumour specific (Figure 1B). Two additional *TOPK* siRNAs were used in HCT116 cells to confirm that the radiosensitisation was not due to an off-target effect (Supplementary Figure 1D). To examine the effect of *TOPK* overexpression on colony formation in HCT116 cells, a CFA was performed, which did not affect radiosensitisation when compared to the empty vector control (Supplementary Figure 1E), suggesting potential differences between endogenous and exogenous *TOPK* expression or the presence of a radioresistance threshold that is not altered by further increase in TOPK levels.

OTS964 is a small molecular compound previously shown to inhibit TOPK kinase activity (Matsuo *et al*, 2014). In order to study the effect of OTS964 on cell viability, the IC_50_ was determined for a panel of cell lines treated with the inhibitor for 72 h. In HAP1 WT and HAP1 *TOPK* cell lines, the IC_50_ was 83 nM and 567 nM, respectively (Supplementary Figure 1F). In addition, the IC_50_ was greater in HFL1 and MRC5 cells than any of the tested cancer cell lines, indicating that cells with high TOPK expression are most sensitive to the inhibitor (Supplementary Figure 2A). We next investigated whether treatment with OTS964 could elicit tumour-specific radiosensitisation comparable to siRNA-mediated TOPK depletion. Treatment of nocodazole-arrested HCT116 cells with 100 nM or 200 nM OTS964 for 4 h is sufficient to inhibit the TOPK substrate motif, HpTGEKP, indicating that TOPK is inhibited under these conditions (Supplementary Figure 2B). Cell lines with an IC_50_ of 60 nM or less were treated with 70–100 nM OTS964 and those with an IC_50_ above 60 nM were treated with 200 nM OTS964. The inhibitor was added 4 h before IR and washed off 24 h after IR. The tested cancer cell lines were radiosensitised (Figure 1C and Supplementary Figure 2C) with SER_10_ values of 2.14 (*P*<0.001) in HeLa, 1.40 (*P*<0.001) in HCT116, 1.42 (*P*<0.001) in DU145, 3.02 (*P*<0.001) in T24, 1.33 (*P*<0.001) in H1299, 1.54 (*P*=0.003) in SQ20B and 1.34 (*P*<0.001) in PC3. The normal cell lines tested were not radiosensitised, with SER_10_ of 0.90 (*P*=0.172) in HFL-1, 1.08 (*P*=0.601) in MRC5 (Figure 1D), 1.04 (*P*=0.817) in HUVEC (umbilical vein epithelium) and 1.00 (*P*=0.466) in HMEC_1 (microvascular epithelium) Supplementary Figure 2D. These experiments suggest that *TOPK* knockdown or kinase inhibition induces tumour-specific radiosensitisation with minimal effects on normal tissue cells.

### TOPK depletion alters cell cycle progression following IR

**Figure 2:**
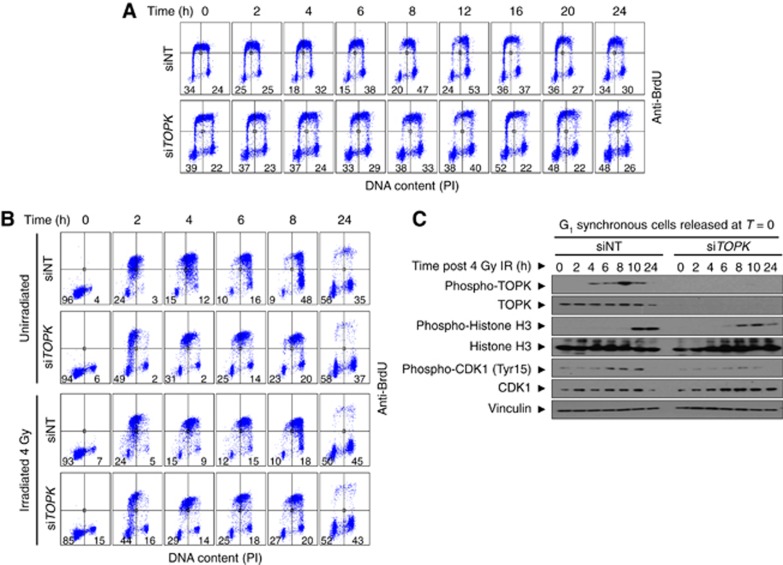
***TOPK* depletion alters cell cycle progression following IR.**Bromodeoxyuridine (BrdU) was added to cells 30 min before fixation and cell cycle stage was assessed by flow cytometry analysis using anti-BrdU and PI staining. The impact of TOPK depletion by siRNA on cell cycle progression was assessed in asynchronous HCT116 cells (**A**) and in HCT116 cells released from double thymidine block (**B**) following 4 Gy irradiation at *T*=0. Numbers indicate the percentage G_1_ population (PI−/BrdU−, lower left quadrant) or G_2_/M population (PI+/BrdU−, lower right quadrant). Early S (BrdU+/PI−, top left quadrant). Late S (BrdU+/PI+, top right quadrant). (**C**) Immunoblotting was used to assess the effect of *TOPK* knockdown on the expression of cell cycle proteins in samples from the experiment described in (**B**). Vinculin was used as a loading control. Data displayed are representative of three independent repeats.

In order to investigate the role of TOPK in the cellular response to IR, a time-course experiment was conducted to determine cell cycle progression of HCT116 cells after 4 Gy IR. Asynchronous cells were irradiated 72 h post siRNA transfection. Cell cycle stage was assessed by flow cytometry analysis using anti-BrdU and PI staining. At 0 h, TOPK depletion caused only slight changes in cell cycle distribution, with a 5% increase in the G_1_ fraction compared to the control, and 3 and 2% decreases in the early S and G_2_/M fractions, respectively (Figure 2A, quantified in Supplementary Table 1). At 2 h after IR, a 12% increase in the si*TOPK* G_1_ population and corresponding decreases of 4 and 3% in the early S, late S and G_2_/M populations, respectively, were observed. The biggest differences were detected at 8 h post IR, with si*TOPK* cells showing an 18% increase in G_1_ phase, a 5% decrease in late S and a 14% decrease in G_2_/M compared with siNT.

The observed increase in G_1_ and corresponding decrease in G_2_/M following IR in TOPK-depleted cells could be due to delay in G_1_ exit, premature mitotic entry, increased cycling rate or a combination of these factors. To distinguish between these possibilities, TOPK-depleted HCT116 cells were synchronised in G_1_ via double-thymidine block, irradiated at 4 Gy and immediately released into fresh culture medium (Figure 2B and Supplementary Table 2). Exit from G_1_ into S-phase was delayed in cells depleted of TOPK when compared with the control (72% decrease in the G_1_ population after 2 h for the control cells, but only a 46% decrease in the G_1_ population after 2 h for the cells depleted of TOPK compared to 0 h). Delayed G_1_ exit in the *TOPK* knockdown cells was independent of IR (Figure 2B). The G_1_ population in the cells depleted of TOPK was nearly triple that of the control at 8 h after release. Correspondingly, there was delayed entry into S phase in irradiated TOPK-depleted cells, with a peak in early S phase at 4 h of only 29%, compared to a higher peak of 48% at 2 h in the control cells. The peak in late S phase was at 8 h for both treatments, but was 45% in the TOPK-depleted cells compared with 66% in the control cells. Cell cycle distribution was comparable at the 24 h time point in the presence or absence of IR for all treatments. These findings are consistent with a delay in G_1_ exit following TOPK depletion.

Markers for cell cycle regulation (cyclin B1 and E1, CDK1 and phospho-CDK1) and mitosis (phosphorylation of histone H3 at Ser_10_) were assessed in G_1_ synchronised HCT116 cells and are presented in Figure 2C and Supplementary Figure 3A. TOPK phosphorylation peaked at 8 h post release in the control cells (Figure 2C). Levels of CDK1 peaked between 6 and 10 h in both the control and TOPK-depleted cells. However, CDK1 phosphorylation (Tyr15) was much reduced in the TOPK-depleted cells, indicating a possible impairment of G_2_/M checkpoint in these cells. Expression of the mitotic markers phospho-histone H3 and cyclin B1 peaked at 10 h post-release in the control cells, reflecting a coordinated progression into mitosis (Figure 2C and Supplementary Figure 3A). In contrast, post-release mitotic entry occurred earlier for cells depleted of TOPK, with H3 phosphorylation and cyclin B1 expression occurring earlier at 6–8 h post release, despite the delayed G_1_ exit (Figure 2C). Cyclin E1 expression confirmed enrichment of G_1_/early S phase cells at the earliest time points (*T*=0 and *T*=2) in the control sample, decreasing post release and reappearing at the latest time point (*T*=24), indicating a return to G_1_. However, cyclin E levels did not decrease as rapidly in the TOPK-depleted cells, consistent with a delay in G_1_ exit. TOPK has been previously shown to have a role in the PI3K/Akt pathway (Ayllon and O'Connor, 2007), the levels of phospho-Akt were therefore investigated after IR and did not show a significant dependency on the levels of TOPK (Supplementary Figure 3B). These results are consistent with both a delay in G_1_ exit and an impaired G_2_/M transition in TOPK-depleted cells.

### TOPK depletion promotes multinucleation and apoptosis after IR

**Figure 3:**
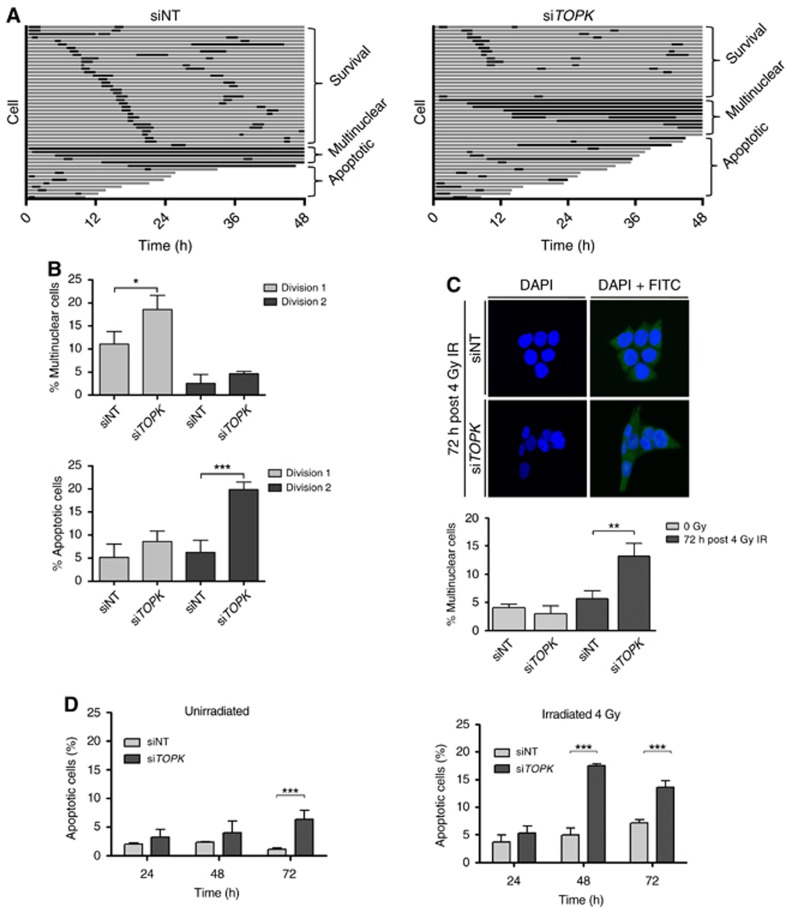
***TOPK* depletion causes multinucleation and apoptosis in HCT116 cells.**Cells were irradiated 72 h post siRNA treatment. (**A**) Live-cell imaging following the fate of 50 HCT116 H2B-mCherry cells for 48 h after 4 Gy IR. Each horizontal bar represents one cell, with interphase shown in grey and mitosis shown in black. Following division, the first daughter cell to undergo division, apoptosis or multinucleation is shown. (**B**) The percentage of cells that apoptosed or became multinuclear during the first or second cell divisions after IR (*n*=3, average of 3 replicates). (**C**) Analysis of IR-induced multinucleation in fixed HCT116 treated cells 72 h after 4 Gy IR. At least 100 cells were counted per condition. Nuclei were stained with the DAPI DNA stain (blue) and FITC was used as a cytoplasmic counterstain (green). (**D**) Cell death in the presence (right panel) and absence (left panel) of 4 Gy irradiation was assessed with Hoechst staining to identify bright apoptotic nuclei in cells transfected with NT or *TOPK* siRNA and irradiated with 4 Gy at *T*=0. Data displayed are representative of three independent experiments and were analysed using unpaired two-sided *t*-tests; *P<0.05, **P<0.01 and ***P<0.001.

Altered cell cycle progression and failure to activate DNA damage checkpoints can result in chromosomal aberrations, mitotic catastrophe, micronucleation, multinucleation and apoptosis (Dillon *et al*, 2014; Visconti *et al*, 2016). To determine the consequences of altered cell cycle progression and G_2_/M arrest in TOPK-depleted cells, we performed live-cell imaging using HCT116 H2B-mCherry cells. The cells were transfected with siNT or si*TOPK*, and after 72 h were exposed to 4 Gy IR. Cells were then imaged every 30 min for 48 h. The fate of 50 cells was determined for each experimental replicate and transitions through mitosis and interphase were recorded (Figure 3A and Supplementary Movies 1–4). There was no significant difference in the length of mitosis, as the first mitosis was 1.9 h in the control cells compared to 1.5 h in the TOPK-depleted cells (*P*=0.231). However, in TOPK-depleted cells there was a significant 8% increase (*P*=0.024) in the number of cells that entered mitosis, attempted cell division and then became multinuclear during the first division (Figure 3B). Furthermore, a nonsignificant 3% increase in apoptosis was observed in TOPK-depleted cells before the first mitosis (*P*=0.705) and a significant 14% increase in apoptosis was observed in the daughter cells prior to the second mitosis (*P*=0.001).

To fully evaluate the impact of this finding at later time-points, we quantified irradiation-induced multinucleation in fixed HCT116 cells following *TOPK* knockdown using DAPI and FITC as nuclear and cytoplasmic stain. *TOPK* knockdown resulted in a significantly higher number of multinucleated cells (*P*<0.01) following irradiation, but no differences were observed in unirradiated cells (Figure 3C). To further investigate the increase in apoptosis in irradiated TOPK-depleted cells, Hoechst and Annexin V assays were used to monitor apoptotic cell death at 24, 48 and 72 h after 4 Gy IR. In both cases, *TOPK* knockdown caused a significant increase in the percentage of apoptotic cells at the latest time points compared to the siNT control (Figure 3D, Supplementary Figure 3C and Supplementary Table 3). The same result was reproducible using si*TOPK*_2 and si*TOPK*_3 siRNAs for the Hoechst assay (Supplementary Figure 3D). Taken together, these data suggest that irradiation of TOPK-depleted cells results in post-mitotic chromosomal instability, multinucleation and ultimately apoptotic cell death.

### TOPK depletion causes chromosomal aberrations in response to IR

In order to test whether TOPK-dependent radiosensitisation was due to erroneous DNA repair, post-IR DNA damage was compared in *TOPK* knockdown cells and control cells using an alkaline Comet assay. No differences in DNA damage were detected at 30 min, 2 or 8 h after 4 Gy IR (Supplementary Figure 3E). The kinetics of γ-H2AX foci formation and resolution was then assessed after 4 Gy IR. Although a small statistically significant decrease in the number of foci was identified in TOPK-depleted cells at 30 min post IR, this was not observed at 4, 8 and 24 h post IR (Supplementary Figure 3F). These results suggest that TOPK does not affect double strand break (DSB) repair.

**Figure 4:**
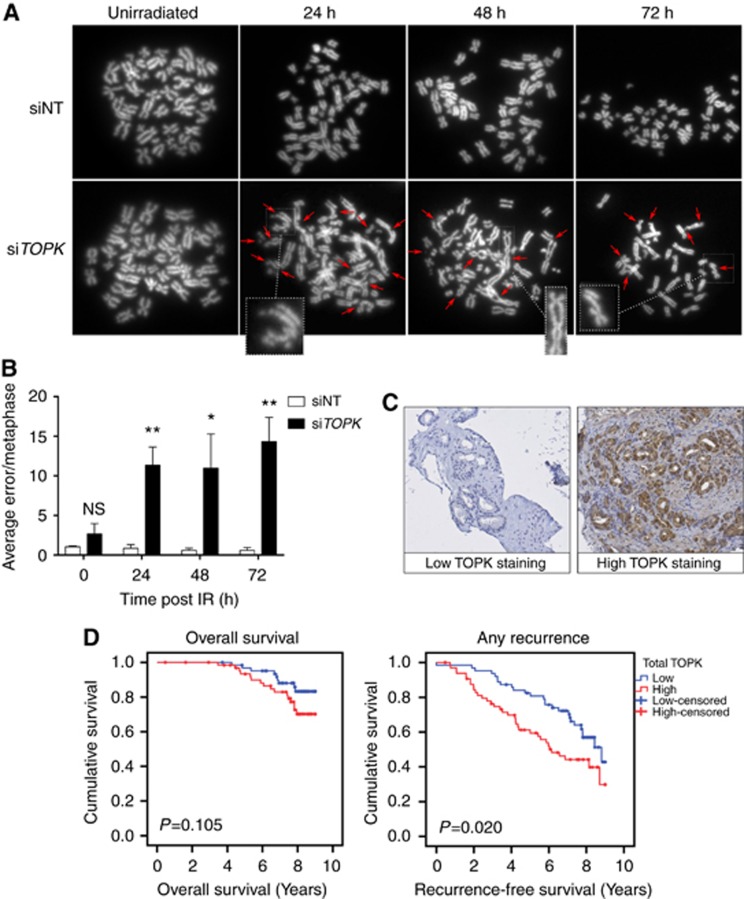
**High TOPK expression correlates with poor outcomes in prostate cancer patients.**(**A**) HCT116 cells were transfected with siRNA and irradiated (4 Gy). Metaphases were collected at 24, 48 and 72 h following irradiation and scored for chromosomal aberrations, with 40 nuclei analysed in each group. Images of representative metaphases include examples of typical errors detected (insets). Arrows indicate chromosomal aberrations. (**B**) Average number of errors per metaphase from three independent experiments *P<0.05 and **P<0.01. (**C**) Representative photomicrographs taken following immunohistochemistry staining with an anti-TOPK antibody, showing non-staining in disease-free prostate epithelium and strong nuclear and cytoplasmic TOPK expression in prostatic carcinoma. (**D**) Kaplan–Meier analysis of TOPK expression and overall survival, and any recurrence in prostate cancer patients following radical radiotherapy (*n*=128). *P*-value from univariate analysis.

To investigate whether *TOPK* knockdown could induce mitotic progression errors, metaphase spreads were prepared in HCT116 cells with siNT or si*TOPK* knockdown and the number of aberrations present per genome were quantified at 24, 48 and 72 h after 4 Gy IR (Figure 4A). Chromosomal aberrations such as breaks, exchanges and fragmentation were significantly greater in TOPK-depleted cells when compared with the control at all time points (Figure 4B).

### TOPK levels affect survival in prostate cancer patients after radiotherapy

To examine the clinical relevance of our findings, the relationship between TOPK expression and therapeutic efficacy was studied in a cohort of 128 prostate cancer patients treated with radical radiotherapy. Over a median (interquartile range) follow-up period of 7.7 (6.9–8.4) years following radical radiotherapy, prostate cancer had recurred in 62/128 (48%) patients, of whom 44/128 (34%) had non-metastatic recurrence and 18/128 (14%) had metastatic recurrence. Deaths from any cause during the follow-up period occurred in 26/128 (20%) patients.

Immunohistochemistry was used to measure TOPK expression from archived prostate biopsies taken at diagnosis and results were analysed with respect to clinical outcomes including recurrence-free survival (RFS) and all-cause mortality. Diagnostic biopsy pathology characteristics of the patient cohort at baseline can be found in Table 1. Samples were ranked according to the levels of TOPK expression and the median of the ranking values was used as a cut-point score for high and low TOPK protein expression (Figure 4C). Multivariate analysis was performed to test whether TOPK expression could be a prognostic factor for post-radiotherapy prostate cancer recurrence as presented in Table 2. High levels of TOPK expression in malignant prostate epithelium in diagnostic prostate biopsies at baseline was significantly associated with a reduced RFS following radical radiotherapy (*P*=0.020). No significant association was observed between levels of TOPK expression and all-cause mortality (Figure 4D). These results indicate that TOPK levels may affect radiotherapy outcome for prostate cancer, and that analysis of TOPK levels might be clinically useful as a prognostic marker when considering treatment with radiotherapy.

## Discussion

TOPK depletion radiosensitised a panel of cancer cell lines derived from lung, prostate, cervical, bladder and laryngeal cancers. This effect was independent of p53 status for the cancer cell lines tested, although most of these cell lines are deficient in p53 (Supplementary Table 4). Radiosensitisation was reproducible with siRNAs targeting multiple *TOPK* sequences and no radiosensitisation was observed in normal cells following *TOPK* knockdown. Comparable results were obtained with the TOPK inhibitor, OTS964. These data indicate that TOPK is a tumour-specific modulator of radiosensitivity.

Depletion of cell cycle regulators can cause radiosensitisation (Ayllon and O'Connor, 2007; Dou *et al*, 2015; Lei *et al*, 2015). In support of this argument, cell cycle regulatory kinases including CDC2, ATM, CDKN2B, CDK5R1 and PLK4 were highly ranked in the screen that identified TOPK (Tiwana *et al*, 2015). Alterations of cell cycle progression in TOPK-depleted cells could be due to delay in G_1_ exit, premature mitotic entry, increased cycling rate or a combination of these factors. Correspondingly, TOPK-depleted synchronous cells that were irradiated in G_1_ and subsequently released had delayed G_1_ exit and an impaired G_2_/M transition. This is consistent with previous findings that TOPK ensures proper arrest at the G_2_/M checkpoint in unirradiated cells (Abe *et al*, 2000; Gaudet *et al*, 2000; Matsumoto *et al*, 2004). Furthermore, TOPK-depleted cells had increased chromosomal aberrations in metaphase, including DNA breaks, chromatid exchanges and DNA fragmentation after IR. The chromosomal aberrations do not seem to arise from a DNA repair defect in TOPK-depleted cells. We propose that these aberrations arise as a consequence of premature mitotic entry, a phenotype that has been observed in ataxia telangiectasia cells, which are deficient in G_2_/M checkpoint activation, but exhibit wild-type levels of chromosomal breaks in G_2_ following IR (Terzoudi *et al*, 2005; Deckbar *et al*, 2007). Failure of cells to arrest at the G_2_/M checkpoint leads to premature chromatin condensation and conversion of unrepaired DSBs into chromosomal breaks, as DSB repair is suppressed during mitosis (Orthwein *et al*, 2014). It is also possible that the chromosomal aberrations arise partly due to inappropriate transition from prophase to metaphase or the inability of C2H2 zinc finger proteins to dissociate from chromatin, as roles for TOPK in these early mitotic activities have been described previously (Park *et al*, 2006; Rizkallah *et al*, 2015).

The studies of both live and fixed cells suggest that a proportion of TOPK-depleted cells attempt mitosis and then become multinuclear during both the first division and during subsequent divisions up to 72 h after IR. Furthermore, a proportion of TOPK-depleted cells divide at least once and then undergo apoptosis during interphase. It is our conjecture that the increases in multinucleation and apoptosis are the consequences of altered cell cycle progression, failure to activate the G_2_/M checkpoint and chromosomal aberrations, causing an increase in radiosensitivity. It is possible that the chromosomal aberrations observed during the first mitosis after IR are inherited by the daughter cells, leading to apoptosis. These findings are consistent with other cell cycle regulation genes that are modulators of radiosensitisation. For example, disruption of the G_2_/M checkpoint protein 14–3–3*σ* promotes early mitotic entry in response to IR, resulting in mitotic catastrophe and cell death (Andreassen *et al*, 2001; Hanahan and Weinberg, 2011). Inhibition of other DNA damage checkpoint proteins such as CHK1 and Wee1 also radiosensitise p53-deficient cells, leading to premature mitotic entry and apoptosis (Garrett and Collins, 2011). TOPK is phosphorylated at Thr9 in G_2_ by cyclin B1/CDK1 to promote mitotic entry and as such is also considered to be a G_2_/M checkpoint protein (Abe *et al*, 2000; Gaudet *et al*, 2000; Matsumoto *et al*, 2004). Therefore, it is likely that there is a common mechanism of radiosensitisation following depletion of G_2_/M checkpoint proteins, including TOPK.

Previous studies have demonstrated a link between high TOPK expression and poor clinical outcomes in different types of cancer, and that TOPK is expressed at low levels in most normal tissues (Rhodes *et al*, 2007; Lei *et al*, 2013, 2015; Park *et al*, 2014; Xiao *et al*, 2015; Ohashi *et al*, 2017). Therefore there is much interest in TOPK as a potential therapeutic target. Our study shows an increased rate of radiotherapy failure in tumours with elevated TOPK expression at baseline, which implies that TOPK expression is an important factor in tumour radioresistance. However, analysis of tumour samples taken as part of a randomised trial of radiotherapy would be required to definitively confirm TOPK expression as a predictive biomarker. Overall survival did not show any significant correlation with levels of TOPK; however, follow-up data did not distinguish between cancer-specific deaths and all-cause deaths and there were few deaths overall. Therefore, it is not possible to state whether TOPK expression influenced overall prostate cancer-specific survival.

Strong correlations between high levels of TOPK and an advanced grade of cancer, metastasis and invasiveness have previously been identified in prostate cancer patients (Brown-Clay *et al*, 2015; Sun *et al*, 2015). Our results support these investigations, revealing a significant association between high TOPK expression and disease recurrence, and add a further level of therapeutic potential by showing these results in the context of radiotherapy. Encouragingly, our study demonstrates that inhibition of TOPK activity is a sensitive and tumour-specific means for enhancing radiosensitivity. As such, the development of small molecule compounds directed against TOPK, such as OTS964, may be a future strategy for enhancing the efficacy of radiotherapy treatment in prostate cancer patients.

## Figures and Tables

**Table 1:** Diagnostic biopsy pathology characteristics of 128 prostate cancer patients at baseline

**Category**	***N*** **(%)**
**Pre-treatment PSA**	
⩽10	46 (35.9)
10.1–20	43 (33.6)
⩾ 20	32 (25)
Unknown	7
**Clinical T stage**	
⩽ 2	78 (60.9)
⩾ 3	42 (32.8)
Unknown	8 (6.3)
**Gleason grade**	
⩽ 6	30 (23.4)
7	83 (64.9)
⩾ 8	15 (11.7)

Abbreviation: PSA=prostate specific antigen.

**Table 2:** Multivariate analysis of clinical prognostic factors for the development of prostate cancer recurrence and any-cause death

	**Any recurrence**		**Any-cause death**	
	**HR (95% CI)**	***P***	**HR (95% CI)**	***P***
**All prostate cancer cases**				
cT stage	1.33 (0.77–2.3)	0.3	0.63 (0.26–1.56)	0.32
PSA	1.07 (0.77–1.48)	0.7	0.86 (0.51–1.46)	0.58
Primary Gleason⩾4	1.72 (1.01–2.93)	0.048	2.2 (0.93–5.2)	0.07
Total TOPK	1.73 (1.02–2.92)	0.04	1.65 (0.72–3.8)	0.24

Abbreviations: CI=confidence interval; HR=hazard ratio; PSA=prostate specific antigen.
