# Supplementary material for: TOPK modulates tumour-specific radiosensitivity and correlates with recurrence after prostate radiotherapy
Source: Br J Cancer. 2017 Jul 4;117(4):503–12. doi: 10.1038/bjc.2017.197 (PMC5558685; doi:10.1038/bjc.2017.197)
Supplement: Supplementary Figure S1 [file bjc2017197x1.ppt]

## Slide 1
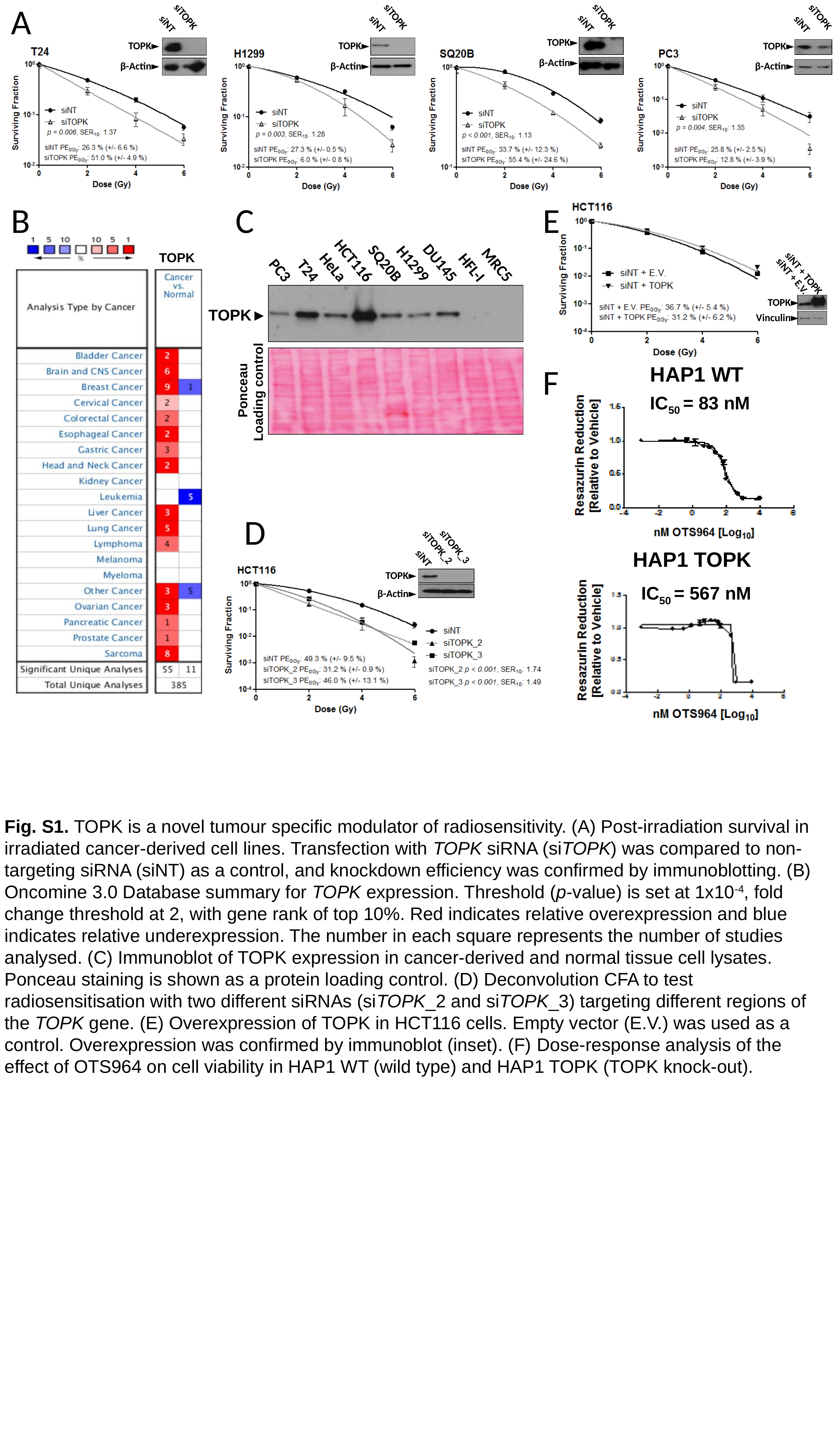

A
siTOPK
siTOPK
siTOPK
siTOPK
siNT
siNT
siNT
siNT
TOPK►
TOPK►
TOPK►
TOPK►
β-Actin►
β-Actin►
β-Actin►
β-Actin►
B
C
E
HCT116
PC3
T24
HeLa
SQ20B
H1299
DU145
HFL-I
MRC5
TOPK►
Ponceau
Loading control
TOPK
siNT + TOPK
siNT + E.V.
TOPK►
Vinculin►
F
HAP1 WT
 IC50 = 83 nM
HAP1 TOPK
 IC50 = 567 nM
D
siTOPK_3
siTOPK_2
siNT
TOPK►
β-Actin►
Fig. S1. TOPK is a novel tumour specific modulator of radiosensitivity. (A) Post-irradiation survival in irradiated cancer-derived cell lines. Transfection with TOPK siRNA (siTOPK) was compared to non-targeting siRNA (siNT) as a control, and knockdown efficiency was confirmed by immunoblotting. (B) Oncomine 3.0 Database summary for TOPK expression. Threshold (p-value) is set at 1x10^-4^, fold change threshold at 2, with gene rank of top 10%. Red indicates relative overexpression and blue indicates relative underexpression. The number in each square represents the number of studies analysed. (C) Immunoblot of TOPK expression in cancer-derived and normal tissue cell lysates. Ponceau staining is shown as a protein loading control. (D) Deconvolution CFA to test radiosensitisation with two different siRNAs (siTOPK_2 and siTOPK_3) targeting different regions of the TOPK gene. (E) Overexpression of TOPK in HCT116 cells. Empty vector (E.V.) was used as a control. Overexpression was confirmed by immunoblot (inset). (F) Dose-response analysis of the effect of OTS964 on cell viability in HAP1 WT (wild type) and HAP1 TOPK (TOPK knock-out).
