# Supplementary material for: TOPK modulates tumour-specific radiosensitivity and correlates with recurrence after prostate radiotherapy
Source: Br J Cancer. 2017 Jul 4;117(4):503–12. doi: 10.1038/bjc.2017.197 (PMC5558685; doi:10.1038/bjc.2017.197)
Supplement: Supplementary Figure S2 [file bjc2017197x2.ppt]

## Slide 1
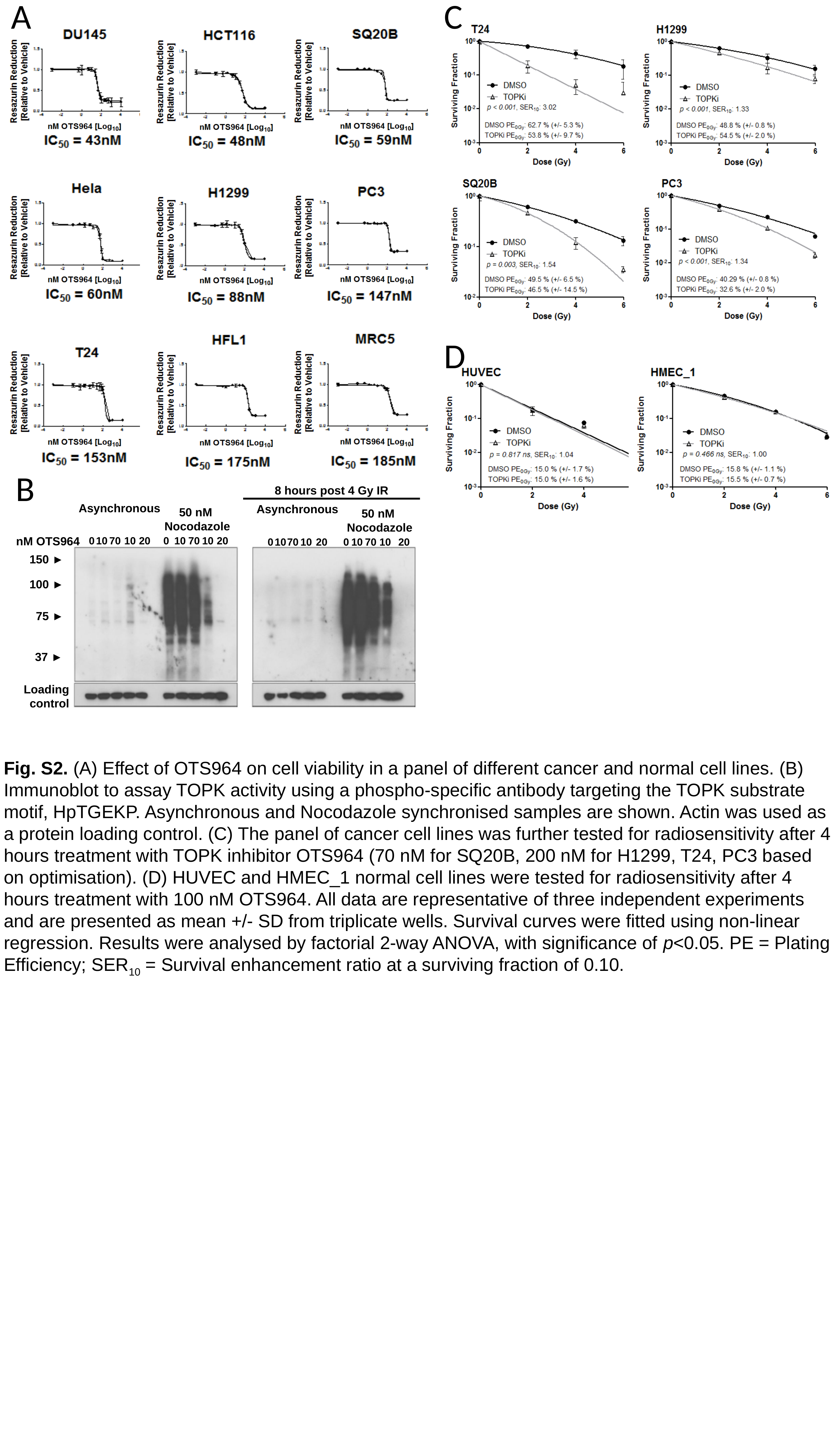

A
C
D
B
8 hours post 4 Gy IR
Asynchronous
Asynchronous
50 nM
Nocodazole
50 nM
Nocodazole
nM OTS964
0
10
70
100
200
0
10
70
100
200
0
10
70
100
200
0
10
70
100
200
150 ►
100 ►
75 ►
37 ►
Loading control
Fig. S2. (A) Effect of OTS964 on cell viability in a panel of different cancer and normal cell lines. (B) Immunoblot to assay TOPK activity using a phospho-specific antibody targeting the TOPK substrate motif, HpTGEKP. Asynchronous and Nocodazole synchronised samples are shown. Actin was used as a protein loading control. (C) The panel of cancer cell lines was further tested for radiosensitivity after 4 hours treatment with TOPK inhibitor OTS964 (70 nM for SQ20B, 200 nM for H1299, T24, PC3 based on optimisation). (D) HUVEC and HMEC_1 normal cell lines were tested for radiosensitivity after 4 hours treatment with 100 nM OTS964. All data are representative of three independent experiments and are presented as mean +/- SD from triplicate wells. Survival curves were fitted using non-linear regression. Results were analysed by factorial 2-way ANOVA, with significance of p<0.05. PE = Plating Efficiency; SER10 = Survival enhancement ratio at a surviving fraction of 0.10.
