# Supplementary material for: TOPK modulates tumour-specific radiosensitivity and correlates with recurrence after prostate radiotherapy
Source: Br J Cancer. 2017 Jul 4;117(4):503–12. doi: 10.1038/bjc.2017.197 (PMC5558685; doi:10.1038/bjc.2017.197)
Supplement: Supplementary Figure S3 [file bjc2017197x3.ppt]

## Slide 1
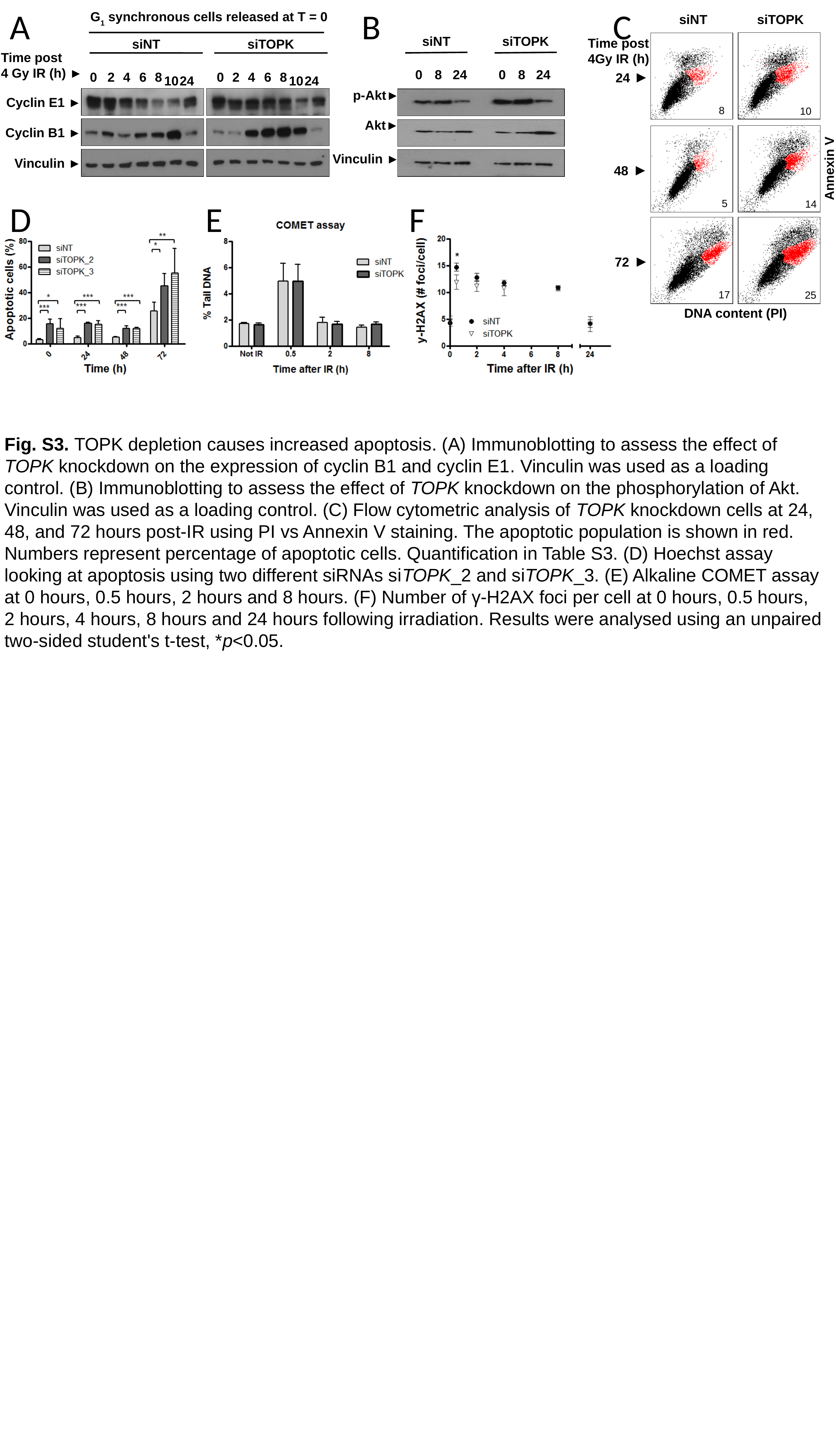

A
B
C
G1 synchronous cells released at T = 0
siNT
siTOPK
Time post
4 Gy IR (h) ►
10
24
10
24
6
8
6
8
0
2
4
0
2
4
Cyclin E1 ►
Cyclin B1 ►
Vinculin ►
siNT
siTOPK
Time post
4Gy IR (h)
10
8
24 ►
14
5
Annexin V
48 ►
17
25
72 ►
DNA content (PI)
siNT
siTOPK
0
8
24
0
8
24
p-Akt►
Akt►
Vinculin ►
D
E
F
Fig. S3. TOPK depletion causes increased apoptosis. (A) Immunoblotting to assess the effect of TOPK knockdown on the expression of cyclin B1 and cyclin E1. Vinculin was used as a loading control. (B) Immunoblotting to assess the effect of TOPK knockdown on the phosphorylation of Akt. Vinculin was used as a loading control. (C) Flow cytometric analysis of TOPK knockdown cells at 24, 48, and 72 hours post-IR using PI vs Annexin V staining. The apoptotic population is shown in red. Numbers represent percentage of apoptotic cells. Quantification in Table S3. (D) Hoechst assay looking at apoptosis using two different siRNAs siTOPK_2 and siTOPK_3. (E) Alkaline COMET assay at 0 hours, 0.5 hours, 2 hours and 8 hours. (F) Number of γ-H2AX foci per cell at 0 hours, 0.5 hours, 2 hours, 4 hours, 8 hours and 24 hours following irradiation. Results were analysed using an unpaired two-sided student's t-test, *p<0.05.
