# Supplementary material for: TOPK modulates tumour-specific radiosensitivity and correlates with recurrence after prostate radiotherapy
Source: Br J Cancer. 2017 Jul 4;117(4):503–12. doi: 10.1038/bjc.2017.197 (PMC5558685; doi:10.1038/bjc.2017.197)
Supplement: Supplementary Movie Legends [file bjc2017197x12.docx]

**File Captions/Figure Legend – Supplementary Movie Files 1-4**

**Time-lapse videos of TOPK depleted HCT116 pH2B-mCherry cells following irradiation**. Brightfield (left panel) and epifluorescence (right panel) images were acquired every 15 minutes using a Nikon Ti-E inverted microscope over a period of 48 hrs.

**Movie File 1:** Failure to progress past metaphase and subsequent nuclear degradation.

**Movie File 2:** Post-mitotic defects in daughter cells culminate in micronucleus formation and apoptosis.

**Movie File 3:** Defective cytokinesis and incomplete separation of daughter cells. Post-mitotic fusion and multinucleation.

**Movie File 4:** Normal mitosis and cell division.
