# Supplementary material for: TOPK modulates tumour-specific radiosensitivity and correlates with recurrence after prostate radiotherapy
Source: Br J Cancer. 2017 Jul 4;117(4):503–12. doi: 10.1038/bjc.2017.197 (PMC5558685; doi:10.1038/bjc.2017.197)
Supplement: Supplementary Figure Legends [file bjc2017197x13.docx]

**Supplementary Figure Legends**

**Supplementary Figure 1.** TOPK is a novel tumour specific modulator of radiosensitivity. (**A**) Post-irradiation survival in irradiated cancer-derived cell lines. Transfection with *TOPK* siRNA (si*TOPK*) was compared to non-targeting siRNA (siNT) as a control, and knockdown efficiency was confirmed by immunoblotting. (**B**) Oncomine 3.0 Database summary for *TOPK* expression. Threshold (*p*-value) is set at 1x10^-4^, fold change threshold at 2, with gene rank of top 10%. Red indicates relative overexpression and blue indicates relative underexpression. The number in each square represents the number of studies analysed. (**C**) Immunoblot of TOPK expression in cancer-derived and normal tissue cell lysates. Ponceau staining is shown as a protein loading control. (**D**) Deconvolution CFA to test radiosensitisation with two different siRNAs (si*TOPK*_2 and si*TOPK*_3) targeting different regions of the *TOPK* gene. (**E**) Overexpression of TOPK in HCT116 cells. Empty vector (E.V.) was used as a control. Overexpression was confirmed by immunoblot (inset). (**F**) Dose-response analysis of the effect of OTS964 on cell viability in HAP1 WT (wild type) and HAP1 TOPK (TOPK knock-out).

**Supplementary Figure 2.** (**A**) Effect of OTS964 on cell viability in a panel of different cancer and normal cell lines. (**B**) Immunoblot to assay TOPK activity using a phospho-specific antibody targeting the TOPK substrate motif, HpTGEKP. Asynchronous and Nocodazole synchronised samples are shown. Actin was used as a protein loading control. (**C**) The panel of cancer cell lines was further tested for radiosensitivity after 4 hours treatment with TOPK inhibitor OTS964 (70 nM for SQ20B, 200 nM for H1299, T24, PC3 based on optimisation). (**D**) HUVEC and HMEC_1 normal cell lines were tested for radiosensitivity after 4 hours treatment with 100 nM OTS964. All data are representative of three independent experiments and are presented as mean +/- SD from triplicate wells. Survival curves were fitted using non-linear regression. Results were analysed by factorial 2-way ANOVA, with significance of *p*<0.05. PE = Plating Efficiency; SER_10_ = Survival enhancement ratio at a surviving fraction of 0.10.

**Supplementary Figure 3.** TOPK depletion causes increased apoptosis. (**A**) Immunoblotting to assess the effect of *TOPK* knockdown on the expression of cyclin B1 and cyclin E1. Vinculin was used as a loading control. (**B**) Immunoblotting to assess the effect of *TOPK* knockdown on the phosphorylation of Akt. Vinculin was used as a loading control. (**C**) Flow cytometric analysis of *TOPK* knockdown cells at 24, 48, and 72 hours post-IR using PI vs Annexin V staining. The apoptotic population is shown in red. Numbers represent percentage of apoptotic cells. Quantification in Table S3. (**D**) Hoechst assay looking at apoptosis using two different siRNAs si*TOPK*_2 and si*TOPK*_3. (**E**) Alkaline COMET assay at 0 hours, 0.5 hours, 2 hours and 8 hours. (**F**) Number of γ-H2AX foci per cell at 0 hours, 0.5 hours, 2 hours, 4 hours, 8 hours and 24 hours following irradiation. Results were analysed using an unpaired two-sided student's t-test, **p*<0.05.
