# Supplementary material for: TOPK modulates tumour-specific radiosensitivity and correlates with recurrence after prostate radiotherapy
Source: Br J Cancer. 2017 Jul 4;117(4):503–12. doi: 10.1038/bjc.2017.197 (PMC5558685; doi:10.1038/bjc.2017.197)
Supplement: Supplementary Information [file bjc2017197x14.docx]

**Supplementary Materials and Methods**

**DNA damage and Hoechst apoptosis assays**. For the comet assay, samples were lysed with an alkaline lysis buffer and were subjected to electrophoresis at 25 V for 25 minutes, at 300 mA. Samples were stained with SYBR Gold. The tail momentum was quantified using Komet 5.5 Software (Andor Technology). To assess γ-H2AX foci, samples were incubated with anti-γ-H2AX antibody followed by AF488 secondary antibody. Cell nuclei were stained with DAPI, and samples were analysed using an InCell Analyzer (GE Healthcare). To quantify apoptosis by Hoechst exclusion, cells were incubated with 20 µg/ml Hoechst for 30-40 minutes, and samples were analysed using the InCell Analyzer (GE Healthcare). To assess apoptosis by Annexin-V, cells were stained using the Annexin-V-FLUOS staining kit (Roche) as indicated by the manufacturer.
